# Genetic Diversity of Tick-Borne Encephalitis Virus in Kyrgyzstan

**DOI:** 10.3390/v18010107

**Published:** 2026-01-13

**Authors:** Leyla H. Shigapova, Irina V. Kozlova, Galya V. Klink, Elena K. Doroshchenko, Olga V. Suntsova, Oksana V. Lisak, Elena I. Shagimardanova, Yuriy P. Dzhioev, Vladimir I. Zlobin, Sergey E. Tkachev

**Affiliations:** 1 Institute of Fundamental Medicine and Biology, Kazan (Volga Region) Federal University, Kazan 420012, Russia; shi-leyla@yandex.ru; 2 Federal State Budgetary Scientific Institution Scientific Centre for Family Health and Human Reproduction, Irkutsk 664003, Russia; diwerhoz@rambler.ru (I.V.K.);; 3 International Laboratory of Statistical and Computational Genomics, National Research University—Higher School of Economics, Moscow 109028, Russia; galkaklink@gmail.com; 4 Genomics and Bio-imaging Core Facility, Moscow 121205, Russia; rjuka@mail.ru; 5 Life Improvement by Future Technologies (LIFT) Center, Moscow 121205, Russia; 6 Loginov Moscow Clinical Scientific Center, Moscow 111394, Russia; 7 Faculty of Preventive Medicine, Irkutsk State Medical University, Irkutsk 664003, Russia; alanir07@mail.ru; 8 National Research Center for Epidemiology and Microbiology Named After Honorary Academician Nikolai Fedorovich Gamaleya of the Ministry of Health of the Russian Federation, Moscow 123098, Russia; vizlobin@mail.ru

**Keywords:** tick-borne encephalitis virus, Kyrgyzstan, subtypes, genetic lineages, *Ixodes persulcatus*

## Abstract

Tick-borne encephalitis virus (TBEV) causes tick-borne encephalitis (TBE), a severe disease of the human central nervous system. Currently, the data on the genetic variants of TBEV in Kyrgyzstan are practically absent. Therefore, the aim of this study was to analyze and describe the genetic diversity of TBEV in this region. The complete genome sequences of seven TBEV strains from the collection of the Scientific Centre for Family Health and Human Reproduction Problems (Irkutsk, Russia) were determined. These strains, isolated from *Ixodes persulcatus* ticks from Kyrgyzstan, were sequenced using the next generation sequencing approach on a MiSeq high-performance sequencer (Illumina, San Diego, CA, USA). A molecular genetic analysis of the obtained sequences, along with sequences of two previously isolated TBEV strains from Kyrgyzstan available in the GenBank database, demonstrated that the Siberian subtype of three genetic lineages (Zausaev, Vasilchenko and Bosnia) is predominantly distributed in Kyrgyzstan. The Far Eastern subtype of TBEV is also present. To date, this location probably represents the southernmost boundary of these TBEV subtypes’ ranges.

## 1. Introduction

Tick-borne encephalitis virus (TBEV), currently classified as *Orthoflavivirus encephalitidis* of the genus *Orthoflavivirus* within the family *Flaviviridae* [1], is the causative agent of tick-borne encephalitis (TBE), a severe disease of the human central nervous system. TBEV has a single-stranded, positive-sense RNA genome of approximately 10.5–11 kb in length. This includes a 5′ type I cap, 5′- and 3′-noncoding regions, and a single open reading frame (ORF), which encodes three structural and seven non-structural proteins [2]. TBE foci have been identified in many European and Asian countries, where up to 12,000 cases of the disease are reported annually. The mortality rate ranges from 0.2% to 20%, depending on the geographic region and, potentially, the viral subtype involved [2].

According to current classification, TBEV is divided into three subtypes: Far Eastern (TBEV-FE), Siberian (TBEV-Sib), and European (TBEV-Eu) [3]. Furthermore, two putative TBEV subtypes, Baikalian (TBEV-Bkl) and Himalayan (TBEV-Him), have been described [4,5]. The TBEV-Eu is primarily distributed across Western, Central and Eastern Europe, as well as the European part of Russia. However, its presence has also been reported in Western and Eastern Siberia and South Korea [2]. TBEV-FE is predominantly found in the Russian Far East and northern China, although isolates of this subtype have been reported in the European part of Russia, Urals, and Western and Eastern Siberia [2]. TBEV-Him has only been detected in the Qinghai-Tibet Plateau in China [5], while TBEV-Bkl has been identified in Eastern Siberia near Lake Baikal and in Northern Mongolia [6]. TBEV-Sib is the most widespread subtype and, with the exception of Central and Western Europe, is present in all regions where TBEV has been detected. In some regions, up to four subtypes have been shown to coexist simultaneously [2]. Five genetic lineages are currently recognized for TBEV-Sib: Zausaev, Vasilchenko, Baltic, Obskaya, and Bosnia [3], each with a distinct geographical distribution pattern. Genetic lineages have also been described for the Far Eastern and European subtypes [2].

Kyrgyzstan, officially the Kyrgyz Republic, is located in Central Asia and borders TBE-endemic regions of Kazakhstan and China. This geographical position creates a risk for the spread of TBE within its territory. The alternation of mountain ranges and intermountain depressions results in exceptional landscape diversity and, as a result, the diversity of arthropods, including TBEV vectors.

Data on the epidemiology and clinical manifestations of TBE in Kyrgyzstan are very fragmentary. Isolated studies indicate that both TBEV and TBE cases have been detected in this territory. For instance, TBEV has previously been identified in local tick populations in Kyrgyzstan [7], and seropositivity for TBEV has been demonstrated in humans and birds [8,9]. However, since the late 1990s, there has been no published data on the spread of TBEV in Kyrgyzstan. In the early 2000s, field studies were conducted to assess the risk level of zoonotic diseases, including TBE. As a result, TBE foci in two areas of the Ala-Archa National Nature Park were identified. A subsequent experiment to collect animals and ticks in these areas resulted in the discovery of TBEV-infected ticks, whose genomes corresponded to the Siberian subtype, as well as the detection of TBEV-specific IgG and IgM in small mammals [10].

Cases of TBE in Kyrgyzstan were reported both in 1976–1981 [11] and more recently [10]. Annually, with the onset of the tick season, local media publish public health guidelines on how to prevent TBE infection (see, for example, [12,13]), which may also indicate the relevance of this issue in the region.

Assessing the spread and studying the specific characteristics of TBEV populations in low-endemic regions is an important objective in virology. Data on the genetic diversity of tick-borne encephalitis virus strains in Kyrgyzstan are virtually absent, which makes this issue particularly interesting. Therefore, the aim of this study was to analyze the genetic diversity of TBEV in Kyrgyzstan.

In this study, we demonstrated for the first time that both the TBEV-Sib and TBEV-FE are present in Kyrgyzstan. TBEV-Sib was represented by at least three genetic lineages: Zausaev, Vasilchenko, and Bosnia. Given that different TBEV subtypes predominantly cause different clinical forms of the disease, these findings are important for predicting disease severity and for guiding future research on tick-borne encephalitis in this region.

## 2. Materials and Methods

### 2.1. TBEV Strains

**Figure 1:**
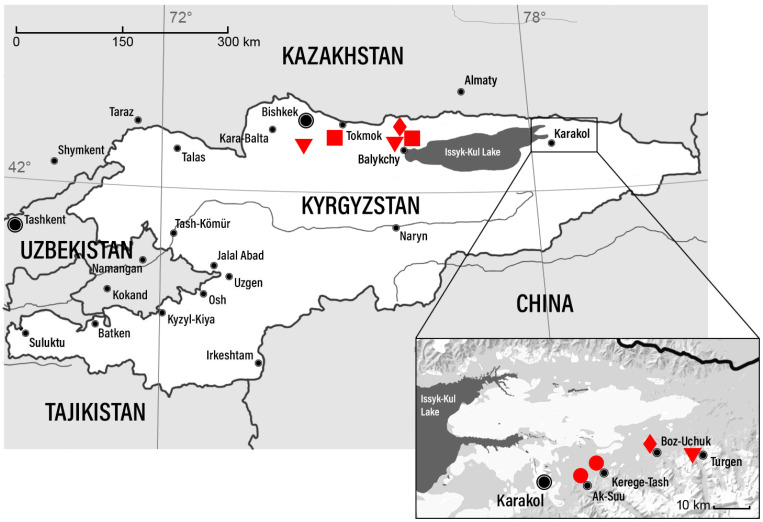
Collection locations and genetic variants of TBEV samples found in Kyrgyzstan. Settlements are marked with black circles. Collection locations and genetic variants of TBEV are marked in red: ● Far-Eastern subtype; Siberian subtype lineages: ■ Zausaev; ▼ Vasilchenko; ◆ Bosnia.

This study employed tick-borne encephalitis virus (TBEV) strains obtained from the collection of the Scientific Centre for Family Health and Human Reproduction Problems, Irkutsk, Russia (Collection No. 478258, https://www.ckp-rf.ru (accessed on 25 November 2025)). The viral isolates originated from *Ixodes persulcatus* ticks collected in Kyrgyzstan in 1986 (Figure 1). Viral stocks were maintained through serial intracerebral passage in outbred white mice (6–7 g in weight). The mice were group-housed (4–6 animals per cage) in standard polycarbonate cages under a conventional light/dark cycle. All animals had *ad libitum* access to food and water. All experimental procedures involving animals were reviewed and approved by the Local Bioethics Committee of the Scientific Centre for Family Health and Human Reproduction Problems (Protocol No. 3.5, 7 April 2021). The study was conducted in full compliance with international guidelines for the care and use of laboratory animals (Directive 2010/63/EU of the European Parliament and of the Council of 22 September 2010).

The map is based on the map of Kyrgyzstan provided by Joint Research Center, ECHO, European Commission (https://commons.wikimedia.org/wiki/File:Kyrgyzstan_Base_Map.png, accessed on 25 November 2025), licensed for free usage and changing under the Creative Commons Attribution 4.0 International (CC-BY-4.0) license.

Information on TBEV strains, collection dates, isolation sources, and collection locations is presented in Table 1.

Brain tissue samples from infected mice were placed in RNA/DNA shield solution (Zymo Research, Irvine, CA, USA) for viral inactivation and to preserve RNA integrity during transport and storage.

### 2.2. TBEV RNA Isolation

RNA was isolated from brain suspensions of TBEV-infected mice using the QIAamp Viral RNA Mini Kit (Qiagen, Hilden, Germany) according to the manufacturer’s protocol.

### 2.3. TBEV Genome Sequencing and Assembly

TBEV genome sequencing and assembly were performed according to the pipeline described in [16]. Briefly, RNA libraries were prepared using the KAPA RNA HyperPrep Kit (Roche, Basel, Switzerland) followed by targeted enrichment with a panel of oligonucleotides designed to correspond to various genome fragments of all known TBEV subtypes and genovariants using SeqCap EZ technology (Roche, Switzerland). The sequencing of the resulting libraries was performed with a MiSeq high-performance sequencer (Illumina, San Diego, CA, USA) using a paired end fragments (2 × 150 bp, 300 total cycles). The quality control of raw reads and data trimming were performed with FastQC (https://www.bioinformatics.babraham.ac.uk/projects/fastqc/, accessed on 25 November 2025) and fastp [17] tools, respectively. The processed reads were mapped against the reference TBEV genome (GenBank: MH645618) using BWA-MEM algorithm within the BWA software package [18]. The resulting data were subjected to sorting and deduplication with the Picard tool (https://broadinstitute.github.io/picard/, accessed on 25 November 2025) followed by the determination of variants (alleles) and consensus sequence generation with the Genome Analysis Toolkit utility package (GATK) (https://gatk.broadinstitute.org/hc/en-us, accessed on 25 November 2025). The coding region sequences of the determined TBEV genomes were submitted to the GenBank database (PX470191–PX470197).

### 2.4. Genetic Analysis

Multiple sequence alignment was performed using the ClustalW method in MEGA 6.0 software [19]. The identity levels of the sequences were calculated using Unipro UGENE v. 52.1 software [20]. Dendrograms were constructed in MEGA 6.0 software [19] using the discrete maximum likelihood method [21]. The most suitable nucleotide substitution model for sequence analysis and dendrogram construction was selected using jModelTest [22] and MEGA 6.0 [19] software. The significance of the dendrogram was assessed using bootstrap analysis with 1000 replicates.

## 3. Results and Discussion

Complete genome sequences of seven TBEV strains were obtained, and the sequences of the genome coding regions were deposited in GenBank under accession numbers PX470191–PX470197. The coding regions were selected for deposition because they were used for further analysis and due to limitations of the sequence assembly method; the non-coding regions of the TBEV genome, particularly the 3′-untranslated region, are variable, and yield inconsistent assemblies depending on the reference sequence used.

Currently, in addition to the complete genome sequences of TBEV strains from Kyrgyzstan presented in this study, the GenBank database (as of November 2025) contains five nucleotide sequences of TBEV genomes and their fragments from this territory. Among them, sequences with accession numbers OR555825 and OR555826 are short sequences of various fragments of the 23-Kyr-26 strain genome, for which the complete genome sequence OR896869 is later presented, while sequence HM641235 is a fragment of the complete genome sequence of KY09 strain (PQ015165) (Table 1).

**Figure 2:**
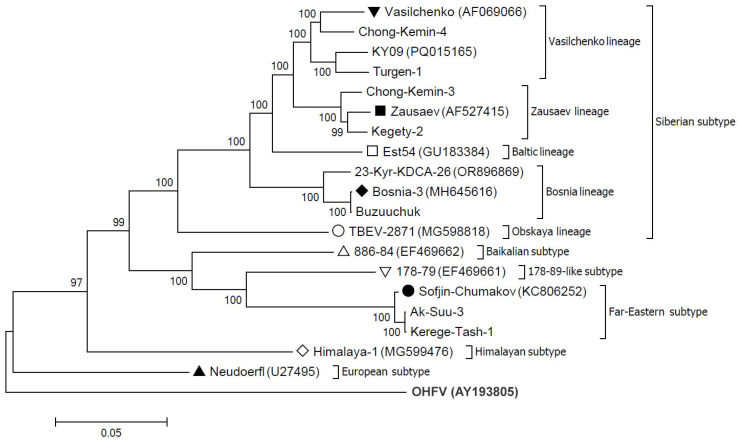
Dendrogram of TBEV strains collected in the Kyrgyzstan, based on the sequences of the genome coding region (133-10377 n.r.) built with the maximum likelihood method. The significance of the constructed dendrogram was estimated by bootstrap analysis with 1000 replicates. GenBank accession numbers of prototype strains and sequences from GenBank database are in brackets. The genome sequence of Omsk hemorrhagic fever virus (outgroup) is marked in bold. The prototype sequences of different TBEV subtypes and genetic linages within the Siberian subtype are marked with: ● Far-Eastern subtype; ▲ European subtype; △ Baikalian subtype; ▽ 178-79-like subtype; ◇ Himalayan subtype; Siberian subtype lineages: ■ Zausaev; ▼ Vasilchenko; ◻ Baltic; ◆ Bosnia; ◯ Obskaya.

A dendrogram was constructed using the sequences of genome coding regions of TBEV strains from this study and related sequences from the GenBank database to assess the genetic diversity of TBEV in Kyrgyzstan (Figure 2).

Dendrogram analysis revealed that the Siberian subtype is predominant in this region, and seven of the nine strains studied belonged to the Siberian subtype. Within the Siberian subtype, three strains belonged to the Vasilchenko genetic lineage (strains Chong-Kemin-4, Turgen-1, and KY09), two strains to the Zausaev genetic lineage (strains Chong-Kemin-3 and Kegety-2), and two strains to the Bosnia genetic lineage (strains 23-Kyr-KDCA-26 and Buzuuchuk). No strains belonging to the Baltic or Obskaya genetic lineages were identified. In addition to the Siberian subtype, two strains of Far Eastern subtype (Ak-Suu-3 and Kerege-Tash-1) were found. The European, Baikalian, or Himalayan subtypes were not detected.

A comparison of the identified genetic variants of the virus with their collection locations (Figure 1) revealed no clear geographical patterns, likely due to the limited number of available TBEV strains. However, the obtained data allowed for a comparison with the genetic diversity of TBEV in Kyrgyzstan neighboring territories.

Previously, only the Vasilchenko but not Zausaev genetic lineage of the TBEV Siberian subtype had been reported in southeastern Kazakhstan [23], although Kazakhstan is located close to Western Siberia, where the Zausaev genetic lineage occurrence is comparable to the Vasilchenko lineage [3].

The discovery of the Buzuuchuk and 23-Kyr-KDCA-26 TBEV strains is of particular interest. Previously, genetically similar TBEV variants classified as the Bosnia genetic lineage of the Siberian subtype had been identified in Bosnia, the Crimean Peninsula [3], and southeastern Kazakhstan [23]. This genetic variant appears to circulate close to the southern border of the TBEV range, as no similar isolates or strains of the virus have been reported further north. Importantly, this genetic variant represents a distinct genetic lineage. An analysis of the identity levels of the genome coding region sequences within and between the Siberian subtype lineages from the GenBank database confirmed that the Bosnia genetic lineage levels of similarity/difference fell within the established boundaries of the genetic lineage division within the Siberian subtype (92–95% nucleotide identity between lineages) (Table 2A,B).

The detection of the Far Eastern subtype in Kyrgyzstan is consistent with data from neighboring regions, particularly China, where this subtype is predominantly detected [24]. However, TBEV strains of the Siberian subtype of the Vasilchenko genetic lineage have also been described in the Xinjiang Uygur Autonomous Region, located near Kyrgyzstan [24], and the TBEV strain belonging to Zausaev genetic lineage was reported in the Inner Mongolia Province [25]. As for other neighboring regions, no data are available on TBEV or TBE in Uzbekistan and Tajikistan.

Thus, two TBEV subtypes, the Siberian and the Far Eastern, were identified in Kyrgyzstan, which likely represents the current southernmost boundary of their geographical range. Information on the distribution of different subtypes in the study area is critical for predicting the disease severity in patients. Previous studies indicate that the Far Eastern subtype predominantly associated with more severe forms of tick-borne encephalitis. In contrast, the Siberian subtype is believed to cause a less severe disease with a tendency to develop chronic infections accompanied by diverse neurological and/or neuropsychiatric symptoms [6]. Nevertheless, given the limited data available, further comprehensive studies of TBEV in this region are required.

## 4. Conclusions

In summary, the analysis of the seven new TBEV strains isolated in this study, together with two previously reported strains from the GenBank database, demonstrates that the Siberian subtype represented by three genetic lineages is predominantly distributed in Kyrgyzstan, while the Far Eastern subtype is also presented. The findings underscore the necessity for systematic epidemiological surveillance of TBEV in this region that could benefit disease control and the prevention of TBEV infection in Kyrgyzstan.

## Figures and Tables

**Table 1:** Information on TBEV strains isolated in Kyrgyzstan.

Strain	Collection Date	Isolation Source	Collection Location	Strain Passage	GenBank Accession No.
Ak-Suu-3	1986	*I. persulcatus*	Ak-Suu village, Ak-Suu District, Issyk-Kul Oblast, Kyrgyzstan	3rd	PX470191
Buzuuchuk	1986	*I. persulcatus*	Boz-Uchuk village, Ak-Suu District, Issyk-Kul Oblast, Kyrgyzstan	3rd	PX470192
Chong-Kemin-3	1986	*I. persulcatus*	Chon-Kemin National Park, Kemin District, Chüy Oblast, Kyrgyzstan	3rd	PX470193
Chong-Kemin-4	1986	*I. persulcatus*	Chon-Kemin National Park, Kemin District, Chüy Oblast, Kyrgyzstan	3rd	PX470194
Kegety-2	1986	*I. persulcatus*	Kegeti village, Chüy District, Chüy Oblast, Kyrgyzstan	3rd	PX470195
Kerege-Tash-1	1986	*I. persulcatus*	Kerege-Tash village, Ak-Suu District, Issyk-Kul Oblast, Kyrgyzstan	3rd	PX470196
Turgen-1	1986	*I. persulcatus*	Turgen village, Ak-Suu District, Issyk-Kul Oblast, Kyrgyzstan	3rd	PX470197
23-Kyr-KDCA-26 * (or 23-Kyr-26 *)	2023	*I. persulcatus*	Kemin District, Chüy Oblast, Kyrgyzstan	n/a	OR555825, OR555826, OR896869 [14]
KY09 * (or Siberian *)	2009	*I. persulcatus*	Ala-Archa National Nature Park, Chüy Oblast, Kyrgyzstan	n/a	HM641235, PQ015165 [10,15]

* Sequences of TBEV strains from the GenBank database (https://www.ncbi.nlm.nih.gov/nucleotide/, accessed on 25 November 2025); n/a—data not available.

**Table 2:** Identity levels of genome coding region (A) and polyprotein (B) sequences between different TBEV-Sib genetic lineages.

**A**					
**Identity Level**		**Genome Coding Region**			
		Zausaev	Vasilchenko	Baltic	Bosnia *
**Genome** **coding** **region**	Zausaev	96–100%	94–95%	93%	92–93%
	Vasilchenko	94–95%	94–100%	92–93%	92–93%
	Baltic	93%	92–93%	96–100%	92–93%
	Bosnia *	92–93%	92–93%	92–93%	98–100%
**B**					
**Identity Level**		**Polyprotein**			
		Zausaev	Vasilchenko	Baltic	Bosnia *
**Polyprotein**	Zausaev	99–100%	96–99%	98%	98%
	Vasilchenko	96–99%	96–100%	98%	98%
	Baltic	98%	98%	99%	99%
	Bosnia *	98%	98%	99%	99%

* Only Buzuuchuk and 23-Kyr-KDCA-26 strains. The cells showing the levels of sequence similarity within a genetic lineage are marked in gray.

## Data Availability

The data obtained in this work are available in the GenBank database under accession numbers PX470191–PX470197.

## Abbreviations

The following abbreviations are used in this manuscript:

ORF	Open reading frame
PCR	Polymerase chain reaction
RT-PCR	Reverse transcription followed by polymerase chain reaction
TBE	Tick-borne encephalitis
TBEV	Tick-borne encephalitis virus
TBEV-Bkl	Baikalian subtype of tick-borne encephalitis virus
TBEV-Eu	European subtype of tick-borne encephalitis virus
TBEV-FE	Far Eastern subtype of tick-borne encephalitis virus
TBEV-Him	Himalayan subtype of tick-borne encephalitis virus
TBEV-Sib	Siberian subtype of tick-borne encephalitis virus
